# Measuring the paradox of self-stigma: psychometric properties of a brief scale

**DOI:** 10.1186/s12991-021-00325-7

**Published:** 2021-01-19

**Authors:** Philippe Golay, Mihaela Moga, Celia Devas, Mélissa Staecheli, Yasmine Poisat, Marie Israël, Caroline Suter, Benedetta Silva, Stéphane Morandi, Pascale Ferrari, Jérôme Favrod, Charles Bonsack

**Affiliations:** 1 Community Psychiatry Service, Department of Psychiatry, Lausanne University Hospital and University of Lausanne, Lausanne, Switzerland; 2 General Psychiatry Service, Treatment and Early Intervention in Psychosis Program (TIPP-Lausanne), Lausanne University Hospital and University of Lausanne, Lausanne, Switzerland; 3 Institute of Psychology, Faculty of Social and Political Science, University of Lausanne, Lausanne, Switzerland; 4 Groupe d’accueil et d’action psychiatrique (GRAAP), Lausanne, Switzerland; 5 La Source, School of Nursing Sciences, HES-SO University of Applied Sciences and Arts of Western Switzerland, Lausanne, Switzerland

**Keywords:** Self-stigma, Mental illness, Questionnaire, Validity, Reliability, French validation

## Abstract

**Background:**

Exposure to public stigma can lead to stereotype endorsement and resignation, which are constructs related to self-stigma. This latter phenomenon has well-documented deleterious consequences for people living with mental illness. Paradoxically, it can also lead to the empowering reactions of righteous anger and coming out proud.

**Aim:**

The aim of this study was to develop and validate a brief tool to measure stereotype endorsement, righteous anger, and non-disclosure across different groups of stigmatized persons. This process was conducted in collaboration with users.

**Method:**

Using focus groups with mental health professionals and people living with mental illness, 72 items were developed to measure various aspects of self-stigma. The Paradox of Self-Stigma scale (PaSS-24) containing 24 items and three subscores (stereotype endorsement, non-disclosure, and righteous anger) resulted from a calibration phase using factor analysis. This structure was cross-validated on an independent sample. Internal consistency, test–retest reliability, and convergent validity were also evaluated.

**Results:**

202 patients were assessed. The PaSS-24 demonstrated good internal validity. Internal consistency, test–retest reliability, and convergent validity estimates were also good.

**Conclusions:**

The PaSS-24 is a short but psychometrically rigorous tool designed to measure self-stigma and related constructs in French language, developed in collaboration with users. The development and validation of the PaSS-24 represent a first step towards implementing and evaluating programs aimed at reducing negative consequences of self-stigma.

## Background

In 1963, Goffman originally defined stigma as an “attribute that is deeply discrediting” and reduces the stigmatized individual from a “whole and usual person to a tainted, discounted one” [1]. Stigma refers to a negative evaluation of a person based on an attribute viewed as different from the norm and can be applied towards individuals from varying backgrounds including race, nation, religion, gender, sexual orientation, psychical characteristics, or health conditions [1, 2]. Stigmatization against people suffering from mental illness is a widespread phenomenon with deleterious effects on at least two important dimensions: public stigma and self-stigma [3–5]. In Corrigan’s view (2005), public stigma and self-stigma share the same cognitive levels of stereotype, prejudice, and discrimination. However, they differ by the fact that public stigma describes “the phenomenon of large groups endorsing stereotypes about and acting against a stigmatized group,” while self-stigma refers to “the loss of self-esteem and self-efficacy that occurs when people internalize the public stigma” [3]. Corrigan later renamed these three stages as “awareness,” “agreement,” and “application,” adding that “harm” occurs only when the person applies the stigma to oneself (2012). Self-stigma lead people to agree to public stigma stereotypes (“People with mental illness are weak”), to apply it to themselves (“I am a weak person because of my mental illness”), and to modify their behavior in consequence (“Why try?”). Both “apply” and “harm” stages belong to the behavioral level. Corrigan’s social-cognitive model of internalized stigma is currently viewed as the prevailing model [6–8] that describes this process. Internalized stigma therefore starts with the awareness of stereotypes associated with one’s condition, followed by agreement with the stereotypes, and the ultimate adoption of the stereotypes on oneself, resulting in lowered self-esteem and self-discrimination [6].

An increasing number of studies have shown that for people living with mental illness, self-stigma can have several negative psychosocial and psychiatric outcomes. A meta-analysis realized by Livingston and Boyd [9] synthesizing the results of 45 studies has found that higher levels of self-stigma were associated with lower levels of hope, self-esteem, empowerment, self-efficacy, quality of life, and social support, as well as with greater severity of psychiatric symptoms and poorer treatment adherence. Another important consequence of self-stigma is the “why try” effect: individuals apply the stereotypes of their health condition to themselves and feel unworthy or incapable to pursue their personal goals [10]. Besides its well-documented negative consequences, research has also outlined a paradox in self-stigma: some people react to it by being righteously angry and becoming more empowered to fight against the injustice experienced [4, 11, 12]. While being constructs related to self-stigma, righteous anger and coming out proud might therefore protect people from its detrimental effects.

People whose self-esteem and self-efficacy are diminished by the internalization of stigma may benefit from interventions targeted towards stigma reduction and from coming out about their condition (Corrigan and Rao 2012). In fact, coming out can reduce the harmful effects of stigma on the quality of life and enhances people’s wellbeing as they feel empowered [13, 14]. There is a great interest to better understand stigma and develop stigma reduction interventions, but little progress has been made regarding the development of instruments that measure the effectiveness of such programs [15]. In a review based on 63 papers, Van Brakel (2006) underlines that the consequences of stigma are similar in different health conditions and it would be possible to develop generic stigma assessment instruments for groups with other attributes than mental health that could be viewed as different from the norm. Stevelink, Wu, Voorend, & van Brakel [16] performed a systematic literature review in order to rate the psychometric properties of the existing self-stigma tools. The results showed that the majority of the actual instruments needed further testing and only two of the 21 scales reviewed received three positive quality ratings: The Child Attitude towards Illness Scale and the Internalized Stigma of Mental Illness [16–18]. The Internalized Stigma of Mental Illness (29-item and 10-item versions) is a widely used questionnaire, measuring alienation, stereotype endorsement, perceived discrimination, social withdrawal, and stigma resistance [17, 19]. Another important scale to mention that measures internalized stigma, shows good psychometric properties, and includes items related to resistance to stigma is the Self-Stigma of Mental Illness Scale [20, 21].

Standardized questionnaires measuring self-stigma are highly needed in French language. Among the existing instruments available to assess stigmatization, we can mention the French versions of King’s et al. Stigma Scale [22, 23] and the Attitudes to Mental Illness 2011 questionnaire, developed as a part of the UK’s anti-stigma campaign Time to Change 2008–2012 [24]. However, these two instruments do not measure self-stigma itself, but rather public stigma and how this latter is perceived by patients. We can also mention the French ISMI scale [19]. The four factors of the ISMI scale are highly correlated and include stereotype endorsement, alienation, social withdrawal, and perceived discrimination.

The aim of this study was to develop and validate with mental health users a short but psychometrically rigorous tool to measure four constructs related to self-stigma in French language: stereotype endorsement, resignation, righteous anger, and non-disclosure. To our knowledge, no other tool did measure successfully this paradoxical dimension of self-stigma that is righteous anger. Another important prerequisite was to create a tool that could be applied across different groups of stigmatized persons (defined by any attribute that could be viewed as different from the norm like gender, sexual orientation, race, religion, and mental or physical health).

## Methods

### Paradox of Self-Stigma scale (PaSS-24)-Item generation

The items of our self-stigma questionnaire were generated using two complementary approaches: literature review and focus groups. Our theoretical model falls within Corrigan’s social-cognitive model of internalized stigma with an emphasis on paradoxical empowerment (Corrigan et al. 2002, 2005, 2012). The objective was to generate a large number of items in order to be able to select the best subset for the final scale.

In the first phase, two psychologists trained in psychometrics and questionnaire development conducted a literature review and identified four domains related to self-stigma (stereotype endorsement, resignation, non-disclosure, and righteous anger) at four levels: awareness (cognitive level, lack of knowledge on mental disorders and their evolution), agreement (affective level, problem of negative attitudes), and application and harm (behavioral level, problem of rejecting, and avoidant behavior [21, 25, 26]. They redacted from scratch a list of 72 items, each based on one of the four domains to stigma at one level identified in the literature review. At the level of “agreement,” *Stereotype endorsement* referred to “the degree to which respondents agree with common stereotypes” about their specific condition (i.e., mental health) (Boyd Ritsher et al. 2003). Typical items of stereotype endorsement were “People with my condition are less useful to society*”* or “People with my condition should be banned from certain jobs.*”* The *righteous anger* described a certain legitimate level of anger in response to stigma. Example of items illustrating this concept are: “The restricted rights of people with my condition is scandalous*”* or *“*I am really fed up with preconceived ideas about my condition.*”* At the level of “harm,” *resignation* implied giving up fighting stigma and believing that there is no point in trying to change the situation. Typical items measuring resignation were *“*What is the point of struggling to have the same rights?*”* or *“*Why bother making any effort when I am inferior to others?*”.* At the level of “application,” *non*-*disclosure* referred to trying to hide one’s medical condition, with items such as: *“*Because of people’s ignorance about my condition, I do not speak to anybody about the problems linked to it*”* or *“*To stop myself from getting into trouble, I avoid situations where my condition might be revealed.*”*

In a second phase, the items were discussed and improved during three focus-group sessions of 2 h each. Focus groups were conducted with mental health professionals, people with mental health problems (acting as experts by experience), and peer practitioners (people with a personal lived experience of mental illness and recovery with standardized training) working together. The focus groups were led by a psychologist and included about ten participants of various age, experience, and gender. The first step was to ask participants to read all items. The second and main step involved discarding, rephrasing, or suggesting new items through a group discussion including every participants. Items were modified each one at a time during this open discussion. The items were modified directly on the screen until validation by all the participants. Participants also insisted on the need of having a neutral response category for the response format. The final questionnaire contained 72 items answered on a 5-point Likert scale: 1 = *“strongly disagree,”* 2 = *“disagree,”* 3 = *“neutral,”* 4 = *“agree,”* and 5 = *“strongly agree.”*

### Participants

A convenience sampling procedure was used. Participants were invited to participate during their hospitalization in different psychiatric hospitals or in other psychiatric residential facilities from three French-speaking Swiss cantons (Fribourg, Vaud, and Neuchâtel). Research assistants (trained master degree psychology students or 6th-year medical students) approached the participants in the presence of their attending nurse and provided them information on the study. Inclusion criteria were to have a psychiatric diagnosis, to be aged between 18 and 65, and to be proficient French speakers. Exclusion criterion was to have a diagnosis of mental retardation. All participants gave written and informed consent. Recruitment took place between September 2017 and October 2019.

## Measures

### The Self-Stigma Scale–Short (SSS-S)

The SSS-S is a 9-item questionnaire designed to measure the extent of self-stigmatization among individuals from various minority groups [27, 28]. Participants are asked to indicate whether they agree or disagree with each of the nine statements on a 4-point Likert scale ranging from *“strongly disagree”* to *“strongly agree.”* The French-language version of the SSS-S was back translated and approved by the original authors. Taken into consideration the feedback received from the participants at the focus groups, we also presented the items on a 5-point Likert scale with an additional neutral response category. In the present study, the internal consistency of the SSS-S was excellent (α = 0.91).

### The general Self-Efficacy Scale (GSE)

The German version of the GSE was developed by Jerusalem and Schwarzer as a 20-item inventory assessing optimistic self-beliefs to cope with difficult demands in life [29]. The scale was later reduced to 10 statements and is considered reliable and valid in numerous studies across different cultures. Typical items are *“I can always manage to solve difficult problems if I try hard enough”* or *“Thanks to my resourcefulness, I know how to handle unforeseen situations.”* Each statement is rated on a Likert scale from 1 (*“not at all true”*) to 4 (*“exactly true”*). The French version of the GSE scale [30] was used in our study and its internal consistency was excellent (*α* = 0.90).

### The Rosenberg Self-Esteem Scale (RSS)

The RSS is the most frequently used instrument to measure self-esteem [31]. It consists of 10 items with a total score ranging from a minimum of 10 to a maximum of 40. Participants respond on a Likert scale by checking one of the four options: *“strongly disagree,”* *“disagree,”* *“agree,”* and *“strongly agree.”* In our study we used the French version of the RSS [32] and its internal consistency was good (*α* = 0.85).

### The Beck Hopelessness Scale (BHS)

The BHS is a widely used questionnaire that measures negative expectations about the future [33]. The inventory is a self-report measure and consists of 20 items scored on a true–false scale. The BHS has a three-factor structure, referring to affective, motivational, and cognitive aspects: feelings about the future, loss of motivation, and future expectations. A total score can be computed and ranges from 0 to 20, with higher scores reflecting higher levels of hopelessness. In the present study, we used the French version [34] of the BHS and its internal consistency was good (*α* = 0.83).

### King’s Stigma Scale–short version (KSS-S)

KSS is a standardized instrument measuring the stigma of mental illness [22]. The questionnaire includes 28 items and 3 subscales: *Discrimination*, *Non*-*Disclosure*, and *Positive Aspects*. The *Discrimination* subscale refers to the negative reactions of others as perceived by the patient. The *Non*-*Disclosure* subscale refers to behaviors adopted to hide being mentally ill in order to avoid discrimination. The *Positive Aspects* of mental illness subscale contains items that describe how people become more accepting and empathetic because of their illness. In our study we used the 9-item French short version of the Stigma scale [23]. This self-report questionnaire has the same three-factor structure as the original version. Participants indicated the extent to which they agree or disagree with each of the 9 statements on a 5-point Likert scale ranging from *“strongly agree”* to *“strongly disagree.”* Considering the three subscores consisted of only three items, their internal consistency could be considered adequate in this study (α *Discrimination* = 0.57; *α Disclosure* = 0.80, *α Positive Aspects* = 0.66).

### World Health Organization Quality of Life, Short Form (WHOQOL‑BREF)

The WHOQOL-BREF [35] is the short version of the WHOQOL-100. It includes 26 Likert-type items, which measure four domains related to quality of life: physical health, psychological health, social relationships, and environment. In this study, we computed and used a total score. In this study, the internal consistency of the WHOQOL-BREF total score was excellent (*α* = 0.93).

## Procedure

The internal validity of the PaSS-24 was assessed in two steps, a calibration phase and a cross-validation phase. For that purpose, the data were randomly split into two independent samples of equal size. The calibration phase aimed to select the best items per subscore on the basis of internal structure. Because the calibration process may capitalize on the chance characteristics of the data (i.e., model overfitting), the proposed structure was then cross-validated on the second sample.

The reliability of the PaSS-24 scores was assessed using a test–retest approach with an interval of between 2 and 14 days. The time interval was kept relatively short in order for the true scores to remain stable across the test–retest interval. Fifty-one participants took part at the retest.

To estimate convergent validity, we studied the relationship between PaSS-24 scores and several other scales. Ninety-six participants completed the other scales than PaSS-24. We hypothesized that the PaSS-24 scores would be positively correlated with the SSS-S and negatively correlated with the GSE. We also hypothesized a positive correlation with the Beck Hopelessness scale and a negative correlation with the King Stigma Scale scores with the exception of the *Positive Aspects* score where we expected a positive correlation. Finally, we expected a negative correlation between the PaSS-24 scores and quality of life (WHOQOL-BREF total score).

## Statistical analysis

### Internal validity

For the calibration phase, we proceeded as follows: for each of the four a priori dimensions, a one-factor model was fitted separately on its respective 18 items. We identified the six items with the lowest loadings of each dimension and discarded them. This process was conducted iteratively one item at a time. This allowed us to reduce the number of items from 72 to 48 and to increase internal consistency. This also ensured that we did not estimate models with a very large number of parameters. Then we estimated a confirmatory factor analysis (CFA) with four dimensions on the remaining items. Because the *Stereotype endorsement* and the *Resignation* factors were highly correlated and very close in terms of content, we decided to merge these two dimensions into one *Stereotype endorsement* factor. In order to reduce the number of items of this new factor from 24 to 12, a one-factor model was estimated. The worst six *Stereotype endorsement* items and worst six *Resignation* items were discarded alternatively following an iterative process. The next step was to estimate a three-factor Exploratory Factor Analysis (EFA) model on the remaining 36 items in order to highlight items with problematic cross-loadings. These items typically load on more than one factor and therefore bias the score interpretation. The worst four items per factor were discarded alternatively in an iterative process. Within the *Stereotype endorsement* factor, we discarded two *Resignation* and two *Stereotype endorsement* items to keep balance between the two concepts. Finally, we estimated a three-factor CFA model on the remaining 24 items (3 dimensions × 8 items). For the sake of parsimony, a simpler one-factor alternative was also estimated. Given the Chi-square difference is not Chi-square distributed with categorical ordinal data, we compared the two alternative final models with a robust Chi-square test.

For the cross-validation phase, we fitted the 24-item three-factor model on the other sample. The single-factor model was also estimated to replicate the model comparison. Finally, and because the cross-validation phase was successful, these two models were also fitted on the whole sample in order to get more precise estimates. Because sample size less than 100–150 could lead to increased over-rejection rates for indices of goodness of fit [36, 37], this final step also allowed us to evaluate model fit on a larger sample.

For CFA and EFA, the models were estimated using a robust weighted least squares estimator with adjustments for the mean and variance (WLSMV). Several indicators of model fit were used: the Root Mean Square Error of Approximation (RMSEA), the Comparison Fit Index (CFI), and the Tucker–Lewis fit Index (TLI). RMSEA values  ≤ 0.06, and CFI and TLI values  ≥ 0.95, were interpreted as good fits, whereas RMSEA values ≤ 0.08, and CFI and TLI values  ≥ 0.90 were considered as indicating satisfactory fit [38].

### Reliability

The reliability of the PaSS-24 subscales was estimated using McDonald’s model-based Omega (ω) [39] and Cronbach’s alpha (α) coefficients. The test–retest reliabilities were estimated using both Pearson and intraclass correlation coefficients using a two-way random-effects model and the absolute agreement definition (ICC  [1, 2]). Reliability coefficients above .70 were considered satisfactory; above .80 were considered good; and above .90 were considered excellent [39, 40].

### Convergent validity

The convergent validity coefficients between the PaSS-24 and the other scales were estimated using Pearson’s correlation coefficients. Under Classical Test Theory, the square root of the score reliabilities acts as an upper bound for validity coefficients. Therefore, the acceptable range is lower than for reliability coefficients. Correlation coefficients between .40 and .60 were considered as good and any values higher than .30 (a medium effect size, according to Cohen [41]) as satisfactory.

All statistical tests were two-tailed, and a significance level was set at *α* = 0.05. All statistical analyses were performed using the Mplus statistical package (version 8.0) and IBM SPSS 25.

## Results

A total of 202 patients participated in the study. Mean age was 42.5 year old and a majority of participants was men. Primary diagnoses based on the International Statistical Classification of Diseases and Related Health Problems 10th Revision (ICD-10) were 30.7% (62) Depression, 29.2% (59) Schizophrenia, 12.9% (26) Mania, 10.4% (21) Personality disorder, 8.4% (17) Alcohol use, 4.0% (8) Anxiety and stress-related disorder, 2.5% (5) Behavioral syndromes associated with physiological disturbances, and 2.0% (4) Drug use. Only 16.3% (33) of the participants were married, the rest were single, divorced, separated, or widowed. Almost 70% (141) of the participants were born in Switzerland and all of them were native or proficient French speakers.

### Internal validity

On the basis of the four separate one-factor models, six items per factor were discarded (*Stereotype endorsement* items 1, 9, 49, 53, 5, and 13; *Righteous Anger* items 66, 30, 58, 42, 62, and 2; *Resignation* items 71, 67, 7, 19, 11, and 3; *Non*-*disclosure* items 20, 60, 28, 36, 56, and 64). Because the *Stereotype endorsement* and *Resignation* factors were highly correlated (*r* = 0.847), the corresponding 24 items were merged and selected in order to create a 12-item *Stereotype endorsement* factor. The following items were discarded: *Resignation* items 23, 39, 47, 27, 31, and 51 and *Stereotype endorsement* items 21, 25, 65, 57, 29, and 61. Based on the three-factor EFA, four items per factor were discarded because of cross-loadings on other factors (*Stereotype endorsement* items 59, 37, 55, and 41; *Righteous Anger* items 10, 26, 70, and 18; *Non*-*disclosure* items 72, 52, 44, and 40). The final 24 items are presented in Table 1 and its English-language translation is available in Appendix 1: Table 5.

**Table 1:** French language version of the PaSS-24

Item		Echelle*
1	Les personnes avec ma condition sont moins utiles à la société	AS
2	La restriction des droits des personnes de ma condition me scandalise	JC
3	En raison de l’ignorance des gens, je ne parle à personne des problèmes liés à ma condition	RD
4	Je me dis « A quoi bon lutter pour avoir les mêmes droits ? »	AS
5	J’en ai vraiment marre des idées reçues sur ma condition	JC
6	En raison des préjugés des gens, je ne parle à personne des problèmes liés à ma condition	RD
7	Certains métiers devraient être interdits aux personnes dans ma condition	AS
8	La méconnaissance du public vis-à-vis de ma condition m’indigne	JC
9	Pour ne pas m’attirer d’ennuis, j’évite les situations où ma condition pourrait être révélée	RD
10	A quoi bon faire des efforts puisque je suis inférieur aux autres	AS
11	Le manque d’informations correctes sur ma condition est scandaleux	JC
12	J’utilise des stratégies pour éviter de parler de ma condition	RD
13	Certaines activités devraient être refusées aux personnes dans ma condition	AS
14	Les stéréotypes sur ma condition me mettent en colère	JC
15	Pour éviter d’être désavantagé, j’utilise des stratégies pour ne pas parler de ma condition	RD
16	Les personnes dans ma condition n’auront jamais une vie heureuse	AS
17	La méconnaissance des médias à l’égard de ma condition est révoltante	JC
18	Pour éviter des remarques désagréables, j’utilise des stratégies pour ne pas parler de ma condition	RD
19	Les personnes dans ma condition devraient rester entre elles	AS
20	Je suis énervé par la façon caricaturale de montrer ma condition à la télévision	JC
21	Pour éviter tout préjudice, je choisis avec qui parler de ma condition	RD
22	Je me suis fait à l’idée que je ne pourrai jamais avoir de vie sociale satisfaisante	AS
23	L’attitude de certaines personnes face à ma condition me révolte	JC
24	Je ne dévoile ma condition à personne pour éviter d’être jugé	RD

*Echelle: AS = approbation des stereotypes/JC = juste colère/RD = réticence à la divulgation

Instructions: Ce questionnaire a pour but d’évaluer votre ressenti par rapport à votre condition de personne malade psychique. Veuillez indiquer pour chaque proposition votre degré d’accord ou de désaccord. Répondez spontanément sans passer trop de temps sur chaque question. Certaines phrases pourront vous sembler étranges, peut être choquantes ou encore répétitives. Ne vous inquiétez pas. Si certaines propositions ne vous correspondent pas du tout, elles peuvent convenir à d’autres personnes. Il n’y a pas de bonnes ou de mauvaises réponses, répondez simplement de la manière qui décrit le mieux vos sentiments

Options de réponse : 1 = *Fortement en désaccord*; 2 = *En désaccord*; 3 = *Neutre*; 4 = *D’accord*; 5 = *Fortement en accord*

The final 24-item three-factor model fit was satisfactory (Table 2; *χ*2 = 416.229; *df* = 249, *p* < .001, RMSEA = 0.082, CFI = 0.936, TLI = 0.929). All items significantly loaded on their respective factors. The single-factor model fit was poor (*χ*2 = 963.118; df = 252, *p* < .001, RMSEA = 0.167, CFI = 0.728, TLI = 0.702). Direct comparison between the two models indicated that the three-factor solution was preferable to the single-factor variant (Δ*χ*2 = 145.130; Δdf = 3; *p* < .001).

**Table 2:** Comparisons of model fit for the PaSS-24

Model	*χ*^2^	Df	*P* value	RMSEA	90% C.I. for RMSEA	CFI	TLI	Robust Chi-Square difference test
Calibration (*N* = 101)								
(a) One-factor model	963.118	252	< .001	0.167	0.156–0.178	0.728	0.702	
(b) Three-factor model	416.229	249	< .001	0.082	0.068–0.095	0.936	0.929	
(a) vs (b)								Δ*χ*^2^ = 145.130, Δdf = 3, *p* < .001
Cross validation (*N* = 101)								
(a) One-factor model	809.874	252	< .001	0.148	0.137–0.160	0.547	0.504	
(b) Three-factor model	379.037	249	< .001	0.072	0.057–0.086	0.894	0.883	
(a) vs (b)								Δ*χ*^2^ = 111.816, Δdf = 3, *p* < .001
Total sample (*N* = 202)								
(a) One-factor model	1579.617	252	<.001	0.161	0.154–0.169	0.649	0.616	
(b) Three-factor model	480.489	249	< .001	0.068	0.059–0.077	0.939	0.932	
(a) vs (b)								Δ*χ*^2^ = 238.534, Δdf = 3, *p* < .001

*df* degree of freedom, *RMSEA* Root Mean Square Error of Approximation, *C.I.* Confidence Interval, *CFI* Comparative Fit Index, *TLI* Tucker–Lewis Index

On the cross-validation sample, the three-factor model fit could be considered as acceptable (*χ*2 = 379.037; df = 249, *p* < .001, RMSEA = 0.072, CFI = 0.894, TLI = 0.883). All items significantly loaded on their respective factors. Again the single-factor model fit was poor (*χ*2 = 809.874; df = 252, *p* < .001, RMSEA = 0.148, CFI = 0.547, TLI = 0.504) and the three-factor solution was preferable to the single-factor variant (Δ*χ*2 = 111.816; Δdf = 3; *p* < .001).

**Fig. 1:**
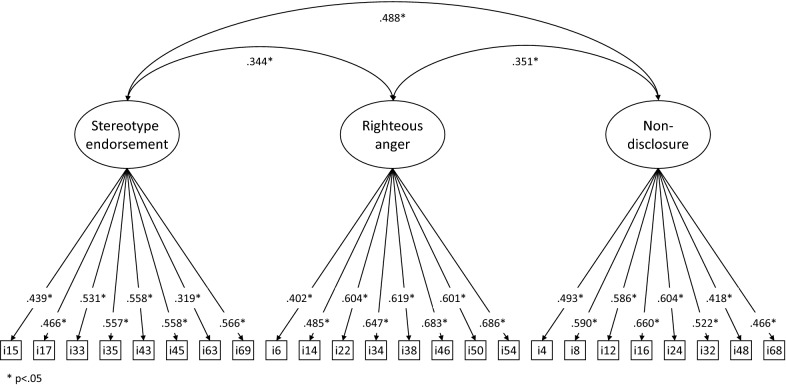
Factor loadings of the PaSS-24

On the total sample, the three-factor model fit was satisfactory (*χ*2 = 480.489; df = 249, *p* < .001, RMSEA = 0.068, CFI = 0.939, TLI = 0.932). However, the single-factor model remained poor (*χ*2 = 1579.617; df = 252, *p* < .001, RMSEA = 0.161, CFI = 0.649, TLI = 0.616) and inferior to the three-factor solution (Δ*χ*2 = 238.534; Δdf = 3; *p* < .001). The loadings of the final model are presented on Fig. 1. The three factors were positively correlated.

### Reliability

Internal consistency estimates (Table 3) were good to excellent. Test–retest reliability estimates were also good. Comparisons between scores from the first and second assessments revealed one significant change. The *Righteous Anger* score was significantly higher during the second assessment (difference = + 2.04 points, t(50) = −3.938, *p* < .001).

**Table 3:** Reliability of the PaSS-24 scores

	Internal Consistency (*N* = 202)		Test–retest reliability (N = 51)		
	McDonald’s ω	Cronbach’s α	Pearson’s r	ICC (2,1)	Standard error of measurement (SEM)
Stereotype endorsement	0.877	0.810	0.879	0.871	2.391
Righteous anger	0.876	0.832	0.828	0.760	2.712
Non-Disclosure	0.914	0.879	0.834	0.829	3.159

* = *p* < .05

### Convergent validity

The correlation between the three PaSS-24 factors and the other scales are presented in Table 4. Most correlation coefficients were substantial, significant, and in the expected direction. Correlations between the *Righteous Anger* score and other scales were typically lower or, in a few instances, not statistically significant.

**Table 4:** Convergent validity of the PaSS-24 scores (*N* = 96)

	PaSS-24		
	Stereotype endorsement	Righteous anger	Non-disclosure
Self-Stigma Short Version (SSS)	.604*	.330*	.573*
General Self-Efficacy Scale (GSE)	− .538*	− .212*	− .297*
Rosenberg Self-Esteem Scale	− .529*	− .179	− .354*
Beck hopelessness scale	.627*	.163	.363*
King stigma scale			
Discrimination	.456*	.500*	.412*
Disclosure	.453*	.348*	.631*
Positive aspects	− .508*	− .074	− .238*
Total Score	.674*	.445*	.628*
WHOQOL quality of life total score	− .645*	− .355*	− .404*

*p < .05

Finally, to facilitate clinical use, normative data on the total sample are presented in Appendix 2: Table 6.

## Discussion

The aim of this study was to develop and validate a French-language instrument measuring self-stigma. The items were generated based on a review of the literature and focus-group sessions including people with mental health problems. They reflected four components related to self-stigma: stereotype endorsement (agree), non-disclosure (apply) resignation (harm), and righteous anger (paradoxical empowerment) (Corrigan et al., 2002, 2005, 2012). The final 24-item self-stigma scale has good psychometric properties and comprises three subscales: *Stereotype endorsement*, *Righteous Anger*, and *Non*-*Disclosure*.

Additionally, the PaSS-24 was developed as a generic inventory for different conditions. The items’ wording was chosen to potentially include other attributes than mental health that could be viewed as different from the norm. We hope it will be validated in English as well as with people from various stigmatized groups.

*Stereotype endorsement* and *Resignation* were almost indistinguishable. Because these two concepts may be at the very heart of self-stigma, this finding was not surprising. The resulting *Stereotype endorsement* factor encompasses these two closely related concepts and may be a simpler score to use.

The three factors were positively correlated. This suggests that *Righteous Anger* does not replace *Stereotype endorsement* or *Non*-*Disclosure,* a phenomenon that would be revealed by a negative correlation between these factors. *Righteous Anger,* which is a legitimate level of anger in response to stigma, was rather associated with these two constructs. The extent to which this could be used as a leverage to fight stigma remains to be further studied.

Our study has several limitations that could be the focus of future studies. First, our study did not consider diagnostics. Second, this study was mainly cross-sectional and a longitudinal design may be used to examine the PaSS-24 sensitivity to change after psychosocial interventions. Third, even if the final model was relatively simple, the 202 participants sample size could not be considered as large and further studies may be useful to replicate our findings concerning the CFA. Fourth, our focus groups did not include a systematic rating of items by the participants. Therefore, content validity indexes could not be computed. Fifth, because of the convenience sampling procedure, refusals or response rate were not documented.

The significance of our results lies in the additional possibility offered to study various aspects of self-stigma in French-speaking populations. This will allow us a better understanding of reducing self-stigma, to monitor and evaluate programs aimed at reducing its negative consequences and to have a significant impact on treatment. Theoretical implications and recommendations for action can be summarized in three ways: individual actions; community responsibilities; and policy implications. Regarding individual actions, mental health professionals should be encouraged to discuss the topic and implications of self-stigma with their patients. The PaSS-24 could be an effective tool to monitor different aspects of self-stigma but also to stimulate discussion around this topic with the patients. Regarding community responsibilities, the negative consequences of self-stigma and the need for specific interventions must be put at the top of the agenda. Awareness campaigns must be developed to ultimately reduce stigma in both the general population and the health professionals. Regarding policy implication, additional regulations are obviously needed to protect patients from stigma and warrant them access to specialized care and adequate treatment. The concept of disclosure and non-disclosure is also in our opinion of paramount importance when developing vocational interventions [42]. Coming out or not is a key question when finding an occupation or protecting the actual professional status if we develop mental health issues. Policy makers could support people with mental illness by implementing measures that help them to conceal their illness if they wish to and by making reasonable adjustments according to anti-discrimination laws when they choose to disclose their condition [5, 43].

## Conclusion

The PaSS-24 is a short, reliable, and valid instrument in French language, developed in close collaboration with users, which measures three constructs related to self-stigma among individuals from different condition like suffering from mental illness. We hope it will stimulate further projects on this topic, have an impact on treatment, and lead us to a better understanding of reducing self-stigma.

## Data Availability

The datasets generated and analyzed during the current study are not publicly available because public archiving of data was not explicitly authorized by the ethic committee. Nevertheless, anonymous data are available from the corresponding author on reasonable request.

## Appendix 1

See Table 5.

**Table 5:** English language version of the PaSS-24

Item		Scale*
1	People with my condition are less useful to society	SS
2	The restricted rights of people with my condition is scandalous	RA
3	Because of people’s ignorance about my condition, I do not speak to anybody about the problems linked to it	ND
4	I tell myself, “What is the point of struggling to have the same rights?”	SS
5	I am really fed up with preconceived ideas about my condition	RA
6	Because of people’s preconceptions, I do not speak to anybody about the problems linked to my condition	ND
7	People with my condition should be banned from certain jobs	SS
8	The public’s lack of knowledge about my condition outrages me	RA
9	To stop myself from getting into trouble, I avoid situations where my condition might be revealed	ND
10	Why bother making any effort when I am inferior to others?	SS
11	The lack of accurate information about my condition is scandalous	RA
12	I use strategies to avoid talking about my condition	ND
13	People with my condition should not be allowed to carry out certain activities	SS
14	The stereotypes about my condition make me angry	RA
15	To avoid being discriminated against, I use strategies not to have to talk about my condition	ND
16	People with my condition will never have a happy life	SS
17	The media’s lack of knowledge about my condition is appalling	RA
18	To avoid disagreeable remarks, I use strategies not to have to talk about my condition	ND
19	People with my condition should stay among themselves	SS
20	I am angry about the way my condition is caricatured on television	RA
21	To avoid any prejudice, I choose who I talk to about my condition	ND
22	I have come to terms with the idea that I will never be able to have a satisfying social life	SS
23	Certain people’s attitudes towards my condition appal me	RA
24	I do not reveal my condition to anybody to avoid being judged	ND

Instructions: This questionnaire aims to evaluate how you feel with regard to your status as a person suffering from a mental condition. Please indicate how much you agree or disagree with each statement. Please respond spontaneously, without taking too much time over each item. Some phrases may seem strange to you, perhaps even shocking or repetitive. Do not worry. If certain statements do not apply to you at all, they will do to other people. There are no good or bad responses; simply give the answer which best describes your feelings. You may answer: 1 = *strongly disagree*; 2 = *disagree*; 3 = *neutral*; 4 = *agree*; 5 = *strongly agree*

*Scale: SS = Stereotype endorsement/; A = Righteous anger; ND = Non-disclosure

## Appendix 2

See Table 6.

**Table 6:** Normative data of the French language PaSS-24 (*N* = 202)

	Stanine 1	Stanine 2	Stanine 3	Stanine 4	Stanine 5	Stanine 6	Stanine 7	Stanine 8	Stanine 9
%	4.0%	6.6%	12.1%	17.5%	19.6%	17.5%	12.1%	6.6%	4.0%
	Very low	Low		Average			High		Very high
Stereotype endorsement	8	9–10	11–13	14–17	18–21	22–24	25–28	29–33	34–40
Righteous anger	8–16	17–20	21–24	25–27	28–31	32–34	35–37	38–39	40
Non-disclosure	8–10	11–15	16–20	21–24	25–28	29–32	33–36	37–39	40

## Abbreviations

BHS: Beck Hopelessness Scale
CFA: Confirmatory factor analysis
CFI: Comparison fit index
EFA: Exploratory Factor Analysis
GSE: General Self-Efficacy Scale
ICC: Intraclass Correlation Coefficient
ICD-10: International Statistical Classification of Diseases and Related Health Problems 10th Revision
KSS-S: King’s Stigma Scale–short version
PaSS-24: Paradox of Self-Stigma scale
RMSEA: Root Mean Square Error of Approximation
RSS: Rosenberg Self-Esteem Scale
SSS-S: Self-Stigma Scale-Short
TLI: Tucker–Lewis fit index
WHOQOL‑BREF: World Health Organization Quality of Life, Short Form
WLSMV: Robust Weighted Least Squares Estimator with Adjustments for the Mean and Variance
